# Spontaneous Splenic Artery Rupture in a Young Adult Without Aneurysmal Disease or Known Risk Factors

**DOI:** 10.7759/cureus.114689

**Published:** 2026-08-17

**Authors:** Jeffrey W Toman, Michael Sauder, Brian Gruber

**Affiliations:** 1 College of Medicine, Northeast Ohio Medical University, Rootstown, USA; 2 General Surgery, St. Elizabeth Youngstown Hospital, Youngstown, USA; 3 Trauma Surgery, St. Elizabeth Youngstown Hospital, Youngstown, USA

**Keywords:** damage control surgery, hemoperitoneum, hemorrhagic shock, nonaneurysmal rupture, splenic artery dissection, splenic artery rupture, visceral artery hemorrhage

## Abstract

Spontaneous splenic artery rupture is a rare but life-threatening cause of intra-abdominal hemorrhage that is most commonly associated with aneurysmal disease, trauma, or underlying vascular pathology. Nonaneurysmal rupture in a young adult without identifiable risk factors, aneurysmal degeneration, or diagnostic vascular findings on computed tomography angiography is exceedingly uncommon and may present a significant diagnostic challenge. We present the case of a previously healthy 27-year-old male who presented with fever, epigastric discomfort, shortness of breath, and lethargy before rapidly deteriorating into hemorrhagic shock and cardiac arrest. Computed tomography angiography demonstrated hemorrhagic ascites without evidence of aneurysmal disease, vascular abnormality, or active contrast extravasation. Emergent exploratory laparotomy revealed spontaneous rupture of the mid-splenic artery with massive hemoperitoneum. The patient underwent damage control distal pancreatectomy and splenectomy with staged re-exploration and intensive postoperative critical care management. Despite profound acidosis with a lowest recorded intraoperative arterial pH of 6.665, coagulopathy, and massive transfusion requirements, the patient survived and was discharged home on hospital day 28. This case highlights the diagnostic difficulty of spontaneous nonaneurysmal splenic artery rupture in the absence of classic risk factors or definitive imaging findings. In patients with unexplained hemoperitoneum and rapid hemodynamic deterioration, clinicians should maintain suspicion for occult visceral artery hemorrhage despite nondiagnostic vascular imaging. Early operative intervention and aggressive resuscitation remain critical for survival.

## Introduction

Spontaneous splenic artery rupture is a rare but life-threatening clinical entity. The most commonly reported underlying etiology is splenic artery aneurysm, which represents approximately 60% of all visceral artery aneurysms and has an estimated prevalence of 0.01%-0.2% in the general population [1]. Splenic artery aneurysm is generally defined as dilation greater than 1.5 times the expected arterial diameter, with treatment often recommended for aneurysms measuring greater than 3 cm. Repair is also indicated for splenic artery aneurysm if symptomatic, growing greater than 0.5 cm per year, associated with pseudoaneurysm, or found in women of childbearing age [2]. Reported risk factors include portal hypertension, systemic hypertension, pregnancy and multiparity, fibromuscular dysplasia, trauma, liver transplantation, and pancreatitis. Splenic artery aneurysms occur more commonly in females and are typically identified in patients older than 50 years of age [3]. Although most splenic artery aneurysms are asymptomatic and incidentally discovered, rupture is a feared complication associated with mortality rates of 20%-36% [4]. Far less commonly, spontaneous splenic artery rupture has been described in association with connective tissue disorders, vasculitis, pseudoaneurysm formation, and isolated arterial dissection involving the celiac axis or splenic artery. Published nonaneurysmal cases commonly involve an identifiable vascular abnormality, inflammatory process, or arteriopathy rather than an entirely unexplained rupture [5-7].

We present the case of a previously healthy 27-year-old male who developed hemorrhagic shock secondary to spontaneous splenic artery rupture without identifiable aneurysmal disease or known predisposing risk factors. Despite advanced imaging, no active contrast extravasation or vascular abnormality was identified preoperatively. Operative findings and surgical pathology did not demonstrate aneurysmal degeneration, pseudoaneurysm, or another definitive underlying vascular pathology. This case highlights the diagnostic challenges and potentially catastrophic clinical course associated with spontaneous splenic artery rupture in the absence of classic risk factors, definitive radiographic findings, or pathologic evidence of aneurysmal disease.

## Case presentation

**Figure 1:**
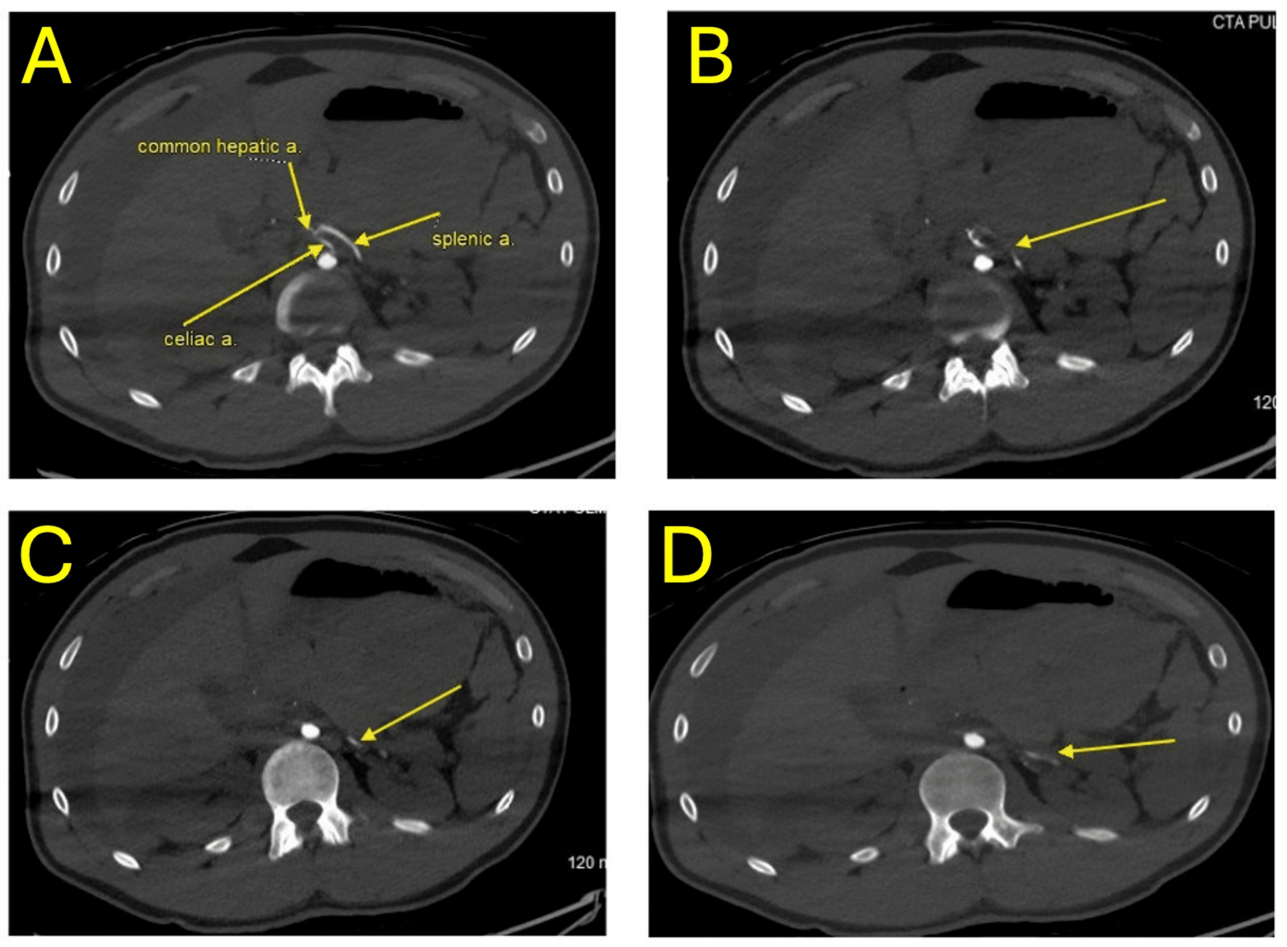
Axial CTA Demonstrating Hemorrhagic Ascites Without an Identifiable Vascular Source Axial computed tomography angiography (CTA) images demonstrating intraperitoneal fluid concerning for hemorrhagic ascites without definitive evidence of active contrast extravasation or visceral artery aneurysm. (A) Celiac axis with labeled common hepatic artery and splenic artery. (B–D) Sequential axial images demonstrating the course of the splenic artery (arrows) without evidence of aneurysmal dilation or active contrast extravasation.

**Figure 2:**
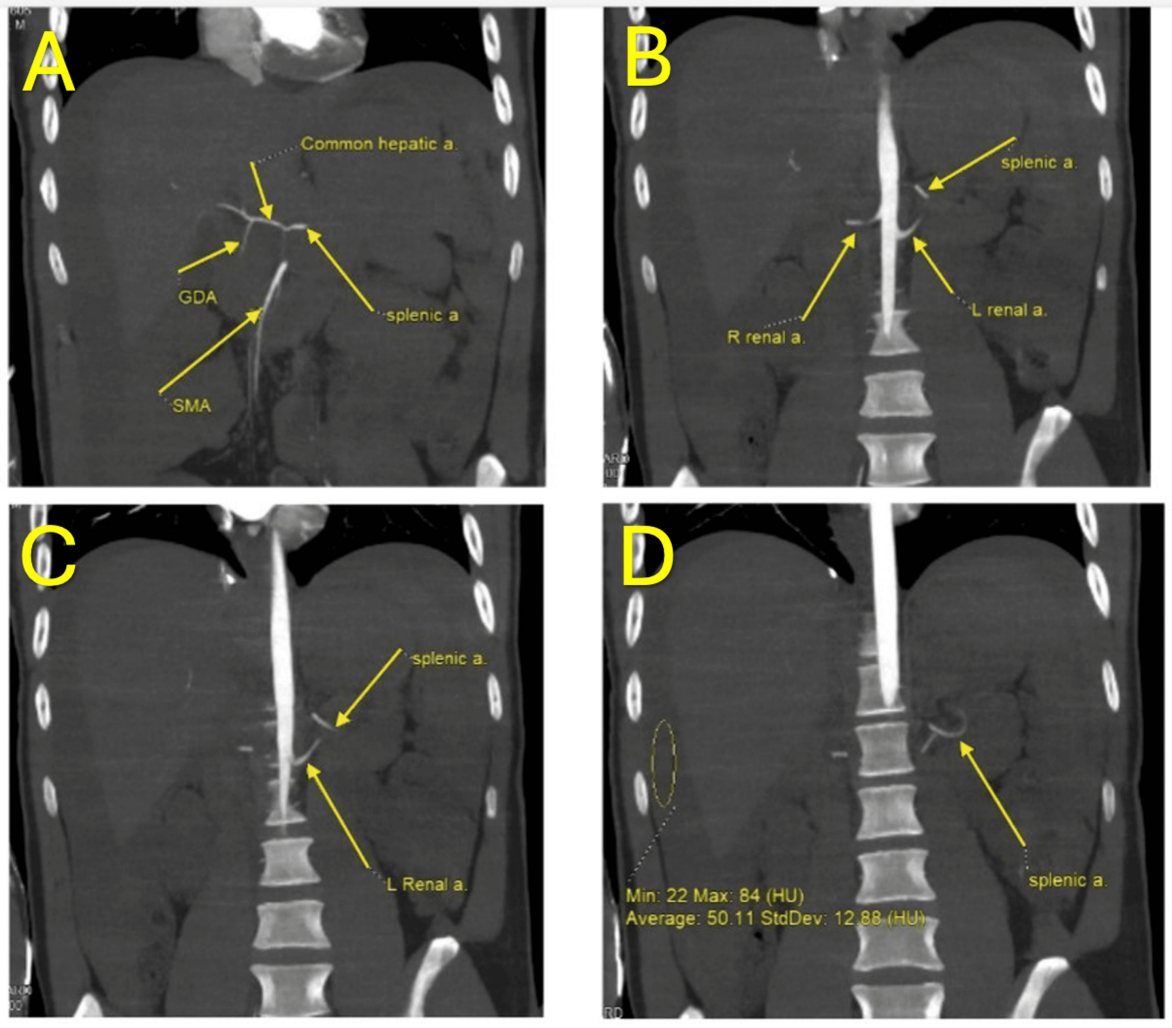
Coronal CTA Demonstrating Hemoperitoneum Without an Identifiable Vascular Source Coronal computed tomography angiography (CTA) images demonstrating intraperitoneal fluid concerning for hemoperitoneum without an identifiable vascular source of hemorrhage. (A) Coronal image of the celiac axis with labeled common hepatic artery, gastroduodenal artery (GDA), superior mesenteric artery (SMA), and splenic artery. (B–D) Sequential coronal images demonstrating the course of the splenic artery (arrows) without evidence of aneurysmal dilation or active contrast extravasation. In panel D, the circled region of intraperitoneal fluid demonstrates an average attenuation of approximately 50 Hounsfield units, supporting hemorrhagic ascites/hemoperitoneum.

A 27-year-old male with a remote history of resected ependymoma presented to the emergency department with fever, headache, epigastric discomfort, shortness of breath, and lethargy. He denied recent trauma, illicit substance use, or use of home medications. Initial laboratory evaluation was notable for a serum lactate level of 11.8 mmol/L (reference range: 0.5-1.9 mmol/L), hemoglobin of 11.8 g/dL (reference range: 12.5-16.5 g/dL), and creatinine of 1.7 mg/dL (reference range: 0.70-1.20 mg/dL). Computed tomography angiography (CTA) of the chest, abdomen, and pelvis demonstrated intra-abdominal fluid with radiodensity of approximately 50 Hounsfield units, concerning for hemorrhagic ascites. No aneurysmal disease or active contrast extravasation was identified on imaging (Figures 1A-1D, 2A-2D). The preoperative differential diagnosis included occult visceral arterial hemorrhage, ruptured visceral artery aneurysm or pseudoaneurysm not visualized on CTA, atraumatic solid organ hemorrhage, and other causes of spontaneous hemoperitoneum. Despite the absence of active contrast extravasation, the combination of hemorrhagic ascites, escalating vasopressor requirement, transfusion requirement, and rapid clinical deterioration raised persistent concern for ongoing intra-abdominal hemorrhage. The patient received aggressive resuscitation with intravenous fluids, vasopressor support, and blood product administration, including 4 L of intravenous crystalloid and 3 units of packed red blood cells; however, he rapidly deteriorated and suffered two episodes of cardiac arrest requiring cardiopulmonary resuscitation. Following sustained return of spontaneous circulation, he was emergently taken to the operating room for exploratory laparotomy.

Prior to entering the peritoneal cavity, blood products were available, and a Cell Saver autologous blood recovery system was prepared. Upon laparotomy, large-volume hemoperitoneum was encountered. All four abdominal quadrants were packed, and the lesser sac was entered. Exploration revealed a large defect within the mid-portion of the splenic artery with pulsatile hemorrhage. Temporary control of bleeding was obtained with proximal clamping of the splenic artery. The spleen and pancreas were otherwise grossly unremarkable. No aneurysmal dilation, pseudoaneurysm, or gross inflammatory process was identified intraoperatively. During the procedure, the patient developed profound coagulopathy, hypothermia, and acidosis consistent with hemorrhagic shock physiology, with a lowest recorded intraoperative arterial pH of 6.665. Estimated blood loss was approximately 6 L. Intraoperative transfusion requirements included 8 units of packed red blood cells, 8 units of fresh frozen plasma, 1 pack of platelets, and 1400 mL of autologous blood via Cell Saver. Given the patient’s critical condition, a damage control approach was chosen. To achieve hemostasis, distal pancreatectomy and splenectomy were performed, with stapled division of the pancreas parenchyma proximal to the location of bleeding on the splenic artery. The left upper quadrant of the abdomen was packed with laparotomy sponges, and temporary closure with abdominal wound vac was applied. The patient was subsequently transferred to the surgical intensive care unit for ongoing resuscitation.

On postoperative day 1, the patient returned to the operating room for second-look laparotomy after developing large-volume hemorrhagic output from the abdominal wound vac. Intra-abdominal exploration demonstrated bleeding from the distal splenic artery stump, which was controlled with suture ligation. Estimated blood loss during the second-look laparotomy was approximately 1 L. The abdomen was again packed with laparotomy sponges and temporarily closed with abdominal wound vac. On postoperative day 3, third-look laparotomy demonstrated viable bowel without further intra-abdominal hemorrhage, and definitive fascial closure was completed. The remainder of the postoperative course was significant for acute respiratory distress syndrome requiring chemical paralysis and mechanical ventilation, with extubation during the first postoperative week; acute renal failure requiring continuous venovenous hemodialysis followed by intermittent hemodialysis; pleural effusion requiring thoracentesis; pancreatic duct leak requiring endoscopic retrograde cholangiopancreatography with stent placement during the third postoperative week; and intra-abdominal abscess requiring percutaneous drainage and intravenous antibiotics. Surgical pathology of the splenic artery demonstrated intact vascular wall with surrounding hemorrhage but no definitive evidence for aneurysmal degeneration. Pathologist examination also found two small accessory splenules measuring less than 1 cm. No other pathologic abnormality was found regarding the pancreas, spleen, or regional lymph node tissue of the specimen.

The patient was ultimately discharged home on hospital day 28 tolerating a regular diet and without ongoing dialysis requirements. At outpatient surgical follow-up approximately two months postoperatively, the percutaneous drain was removed, and the patient reported improving appetite, weight gain, and activity tolerance. At approximately four months postoperatively, the pancreatic duct stent was successfully removed without recurrent abdominal pain or hemorrhagic complications.

Written informed consent was obtained from the patient for publication of this case report.

## Discussion

The splenic artery is the visceral artery most commonly affected by aneurysmal disease. True aneurysms involve dilation of all three layers of the arterial wall, whereas pseudoaneurysms result from a focal defect in the intima and media, leading to a collection of blood contained by the adventitia. True splenic artery aneurysms are typically asymptomatic and are often discovered incidentally. Recognized risk factors include portal hypertension, systemic hypertension, pregnancy, atherosclerosis, and connective tissue disorders. Although rupture is uncommon, it is associated with substantial mortality [8]. Risk factors for splenic artery pseudoaneurysm include trauma, pancreatitis, peptic ulcer disease, and prior medical intervention [9]. In contrast, spontaneous splenic artery rupture in the absence of aneurysmal disease is exceedingly rare and remains poorly characterized in the literature. Notably, the patient in the present case had none of the recognized risk factors for true aneurysm or pseudoaneurysm and no relevant family history or clinical stigmata suggestive of an underlying connective tissue disorder.

Several mechanisms for nonaneurysmal splenic artery rupture have been described, including isolated splenic artery dissection, vasculitis, fibrodysplastic disease, and segmental arterial mediolysis (SAM). Case reports describing splenic artery dissection without aneurysm formation demonstrate that catastrophic hemorrhage may occur despite the absence of pre-existing vascular dilation or radiographic abnormality [5-7]. In contrast to previously reported nonaneurysmal cases associated with dissection, vasculitic features, or other arteriopathic changes, the present case lacked a clear radiographic, intraoperative, or histopathologic explanation for rupture [5-7]. SAM is an additional important consideration in spontaneous visceral artery rupture and is characterized by noninflammatory degeneration of the arterial media that may predispose to dissection and vessel rupture [10,11]. However, definitive diagnosis of these entities requires histopathologic evidence, which was not identified in the present case. Surgical pathology demonstrated an intact vascular wall with surrounding hemorrhage but no definitive aneurysmal degeneration, supporting the unusual nature of this presentation in an otherwise healthy young patient without known predisposing risk factors. Taken together, the absence of aneurysmal degeneration, pseudoaneurysm, traumatic mechanism, pancreatitis, gross inflammatory process, or pathologic evidence of SAM or vasculitis limited the ability to assign a specific etiology.

The diagnosis of spontaneous splenic artery rupture can be particularly challenging due to its nonspecific presentation and potentially nondiagnostic imaging findings. Our patient initially presented with constitutional and vague abdominal symptoms rather than classic signs of hemorrhagic shock. Although CTA demonstrated intra-abdominal fluid with attenuation concerning for hemorrhagic ascites, no aneurysmal disease or active contrast extravasation was identified. The absence of active extravasation on CTA, however, does not exclude clinically significant arterial hemorrhage. Prior studies evaluating splenic and retroperitoneal bleeding have demonstrated that CTA sensitivity for active hemorrhage is imperfect, particularly in cases of intermittent bleeding or temporary tamponade [12,13]. Similarly, contained vascular injuries may only become apparent during specific imaging phases or after further hemodynamic deterioration [12]. In the present case, rapid clinical decompensation despite resuscitative efforts ultimately prompted emergent operative exploration, which proved lifesaving. This case therefore highlights the importance of maintaining suspicion for occult intra-abdominal hemorrhage even in the setting of nondiagnostic vascular imaging.

Given the patient’s profound physiologic derangement, management required a damage control surgical approach focused on rapid hemorrhage control and staged resuscitation. During exploratory laparotomy, the patient developed severe acidosis, coagulopathy, and hypothermia consistent with the lethal triad associated with massive hemorrhage. Damage control laparotomy with temporary abdominal closure allowed for abbreviated operative intervention while ongoing correction of metabolic and hemodynamic instability was performed in the intensive care unit. Planned re-exploration subsequently permitted definitive control of the splenic artery stump and delayed fascial closure after physiologic stabilization. Damage control principles are well established in trauma surgery and have increasingly been applied to nontraumatic intra-abdominal hemorrhage when severe physiologic compromise is present [14,15]. The patient’s survival despite profound shock physiology, including an intraoperative pH of 6.665 and massive transfusion requirements, underscores the importance of timely operative intervention and multidisciplinary critical care management in catastrophic visceral artery hemorrhage.

This report has several limitations. First, as a single case report, it cannot establish the incidence, optimal diagnostic approach, or best management strategy for spontaneous nonaneurysmal splenic artery rupture. Second, although the patient lacked recognized risk factors and surgical pathology did not demonstrate aneurysmal degeneration, vasculitis, SAM, or another definitive vascular abnormality, an occult or transient arteriopathy cannot be completely excluded. Third, the absence of active contrast extravasation on CTA limits the ability to correlate the precise site of preoperative imaging abnormality with the intraoperative rupture site. Therefore, this case is best interpreted as a rare presentation of spontaneous nonaneurysmal splenic artery rupture without an identifiable cause after available clinical, radiographic, operative, and pathologic evaluation, rather than definitive proof of a truly idiopathic mechanism.

A high index of suspicion is therefore essential when evaluating patients with unexplained hemoperitoneum or rapid hemodynamic deterioration, even in the absence of trauma, aneurysmal disease, or definitive imaging findings. Although spontaneous nonaneurysmal splenic artery rupture remains rare, delayed recognition may carry devastating consequences. Early operative exploration should be strongly considered in unstable patients when occult visceral artery hemorrhage remains a concern.

## Conclusions

Spontaneous splenic artery rupture is a rare but life-threatening cause of intra-abdominal hemorrhage that may occur even in the absence of aneurysmal disease, identifiable vascular pathology, or classic predisposing risk factors. This case adds to the limited literature describing spontaneous nonaneurysmal splenic artery rupture and highlights the diagnostic difficulty associated with nonspecific presentation and initially nondiagnostic vascular imaging findings. Although conclusions from a single case report should be interpreted cautiously, unexplained hemoperitoneum with rapid hemodynamic deterioration should prompt consideration of occult visceral artery hemorrhage, even when definitive radiographic evidence of active bleeding is absent. Prompt recognition, aggressive resuscitation, and timely operative intervention may be critical in selected unstable patients with catastrophic visceral artery hemorrhage.
